# Developing a health system approach to disaster management: A qualitative analysis of the core literature to complement the WHO Toolkit for assessing health-system capacity for crisis management

**DOI:** 10.1371/5028b6037259a

**Published:** 2012-08-22

**Authors:** Claire Bayntun, Gerald Rockenschaub, Virginia Murray

**Affiliations:** WHO Collaborating Centre for Mass Gatherings and Extreme Events, Health Protection Agency, London; World Health Organization (WHO) Regional office for Europe, Copenhagen, Denmark; Head of Extreme Events and Health Protection, Health Protection Agency, London UK

## Abstract

BACKGROUND The World Health Organisation's (WHO) sixty-fourth World Health Assembly in May 2011 adopted a resolution on ‘strengthening national health emergency and disaster management capacities and resilience of health systems’. Disaster management is a topical issue globally and countries are being encouraged to improve their disaster preparedness, along with growing international commitment to strengthening health systems. Lessons identified from disasters have not been effectively collated; essential experience is forgotten. METHODS This paper describes the analysis of the worldwide experience of disasters through a health systems approach. A systematic search of the core literature from January 2000 to November 2011 was conducted. Components drawn from the WHO’s Global assessment of national health sector emergency preparedness and response baseline survey were combined with WHO’s six health system building blocks (or levers) to act as the initial analysis anchors, with a further grounded theory qualitative analysis of the literature allowing the identification of emerging themes and insights. The priority areas identified by this literature review were then compared with the topics covered by the new expert-consensus-derived Toolkit for assessing health-system capacity for crisis management developed by the WHO Regional Office for Europe. FINDINGS 143 publications identified from a literature search were analysed and appraised. Themes and examples from the literature demonstrate how health system strengthening should contribute to disaster management. Priority areas under-represented in the WHO Toolkit and identified by the qualitative analysis are discussed. INTERPRETATION Collation and analysis of the disaster management literature identifies how health system strengthening can promote resilience and efficient recovery in the face of disasters. These findings support and complement the WHO Toolkit. Countries can use the literature evidence with the WHO Toolkit to assess their disaster management capacities and identify priorities for strengthening their health system. Citation: Bayntun C, Rockenschaub G, Murray V. Developing a health system approach to disaster management: A qualitative analysis of the core literature to complement the WHO Toolkit for assessing health-system capacity for crisis management. PLOS Currents Disasters. 2012 Aug 22. doi: 10.1371/5028b6037259a.

## Introduction

Are we prepared for disasters?

Each disaster and its context are different, yet many share similar health sector vulnerabilities and thus common disaster management practices and policies can be built in to the health system to create resilience – the ‘all-hazards’ approach. Four recent major disasters exemplify the need for strengthened health systems.

The earthquake in Haiti, 12^th^ Jan 2010, left 1.5 million homeless and killed 149,095 people of which 6300 died in a potentially preventable cholera outbreak which infected a further 450,000 residents.^1^ Haiti lacked an effective health system prior to the earthquake and national authorities were not equipped to manage relief or recovery priorities when the disaster occurred.^2^ Governance structures were destroyed and the required services, health workers, surveillance, resources, funding and (attempts at) coordination were provided almost completely by international organisations, creating its own set of complications and delaying investments into the health system.^3^

Floods in Pakistan, July to August 2010, affected 20 million people and destroyed health facilities. In spite of the public health challenges, previous disaster management investment in southern Punjab region allowed for effective evacuations and saved lives.^4^

The famine in the Horn of Africa in 2011 affected 10 million people across several countries. Large population displacement created additional public health challenges to areas that have poorly developed health systems and lack disaster preparedness. Immediate priorities included - provision of water, sanitation, and shelter; trained staff to address widespread acute malnutrition; surveillance for outbreaks; vaccine programmes for preventable diseases; funding; and inter-agency coordination.^5^ These reflect the different components of a health system.

The earthquake and tsunami in Japan, 11^th^ March 2011, caused destruction of healthcare facilities; initial shortages of food, water, fuel, aid materials and rescue teams to the affected rural population; 400,000 people were evacuated to shelters with no heating in freezing temperatures. However, Japan had invested in disaster management and had created a more resilient health system which continued to function in spite of the challenges.^6^

WHO describes public health threats as: ‘new or newly emerging diseases, the accidental release or deliberate use of biological, chemical or radio-nuclear agents, natural disasters, human-made disasters, complex emergencies, conflicts and other events with a potentially catastrophic impact on human health’.^7^ Public health emergency preparedness has been described as “the capability of the public health and health-care systems, communities, and individuals to prevent, protect against, quickly respond to, and recover from health emergencies, particularly those whose scale, timing, or unpredictability threatens to overwhelm routine capabilities”.^8^ The inclusion of disaster ‘prevention’ reflects the public health perspective which is proposed here, whereas preparedness in ‘emergency management’ is limited to the actions to anticipate and build response capacity.

Health system can be defined as ‘comprising all the resources, organizations and institutions that are devoted to producing interdependent actions aimed principally at improving, maintaining or restoring health’.^7^ WHO describes it as being composed of six building blocks, each involving public health – 1) service delivery, 2) health workers, 3) health information, 4) medical products, technology and vaccines, 5) health finance, and 6) governance and leadership. This paper describes these components as ‘levers’ to encapsulate the complex and dynamic nature of health system’s functioning reflecting the inter-dependent movements of its levers.

Historically, different aspects of disaster management have been considered and discussed in isolation. However, the ‘multi-disciplinary health systems’ approach to disaster management suggests that each component of a health system needs resilience to threats built in to its structure. In this way, the whole health system can be strengthened to meet the demands of any type of disaster, enabling a coordinated, rapid and effective response and recovery.

The WHO Regional Office for Europe has adopted health system strengthening as the approach to support emergency preparedness and enhancement of crisis management capacities of member states. In response to international requests, a practical, action oriented Toolkit was developed and refined in a series of expert consultations.

The Toolkit for assessing health-system capacity for crisis management was piloted in 2007 and 2008 in an earlier version in multidisciplinary country missions to Armenia, Azerbaijan and the Republic of Moldova,^9^ with the respective assessment reports published on the WHO/Europe website. Based on the feedback and experience, the Toolkit was adapted and fine-tuned.

The Toolkit consists of a “User manual” and the “Assessment form” and is structured using the WHO health systems framework - subcategorized into 16 key components and 51 essential attributes - to facilitate a structured and reproducible assessment of the preparedness of health systems, based on defined indicators.

The final revised version, published in January 2012, was used to prepare a country report for Turkey,^10^ and Croatia. The methodology has been applied for targeted assessments to evaluate public health preparedness for an increased influx of migrants following the North Africa Crisis in assessment missions to Malta,^11^ Italy (Lampedusa)^12^ and Greece (Evros Region).^13^ The scope of the application of the assessment tool has been broadened towards a self-assessment methodology for countries to identify gaps in health system capacities and to measure progress towards indicators.

A report using the WHO Toolkit to evaluate England’s health system arrangements to deal with crises, risk prevention and mitigation initiatives was submitted in January 2012. The findings demonstrate the advantages of the largely integrated nature of the National Health Service (NHS) in England in regard to disaster management, working in conjunction with the structured response system that involves multi-sector collaboration and coordination.

In spite of this progress there remains a lack of evidence or collated evidence about the use of a health system-wide approach to disaster management; it has not yet received attention in the disaster-related core literature.^14^ Lessons identified from disasters are not readily accessible resulting in disaster management experience being forgotten. Important frameworks exist to guide disaster management, such as the United Nations International Strategy for Disaster Reduction’s Hyogo Framework for Action,^15^ and the WHO Regional Office for Europe Toolkit, which are developed through expert consultation to create a consensus set of guidelines and checklist. This paper contributes an alternative approach, drawing from the collated actual worldwide experience of disasters in the published in the literature. The qualitative analysis of the evidence and experience found in the literature supports the Toolkit’s objective to build resilience across the health system levers for an all-hazards approach to disaster management.

## Methods

**Analysis- The worldwide experience of disasters drawn from the core literature.** A systematic review was conducted of the core literature in Medline and Embase databases published between 1st January 2000 and 18th November 2011. Search terms comprised of disaster terms combined with the components of the health system – full details of the systematic review and the documents identified are reported elsewhere.^16^ Content analyses and evidence assessment (using Harden’s quality appraisal tool 17 ) was completed on each of the 143 documents identified by the search. An additional qualitative analysis identified the strategies for developing a health systems approach to disaster management.^14^ Priorities identified by the analysis are compared with those used in the expert-consensus-derived WHO Toolkit, and the relevant experience found in the literature discussed.

An initial content analysis was conducted on each document identified by the systematic review^14^ using a matrix combining the WHO’s six levers of health systems with components drawn from the WHO’s Global assessment of national health sector emergency preparedness and response,^18^ WHO’s baseline preparedness survey of Member States.

Further to completing the content analysis matrix, a grounded theory qualitative analysis approach was employed.^19^ Extracts were collated according to each health system lever area as they combined with components drawn from WHO’s baseline survey – each box of the matrix acting as an ‘anchor’ from which concepts and categories were developed. Thus the analysis is structured while allowing the reviewed papers to deliver the emerging themes and examples - the collated worldwide experience thus indicating how health system strengthening can contribute to disaster management.

The range and number of information-rich studies included limited the risk of any disaster management theme being under-represented by the process. Additionally, while all documents analysed were published in the peer-reviewed core literature, the review encompasses a range of materials, including case reports. Thus the type and quality of ‘evidence’ is broad, and comments detailing the type of evidence formed part of the quality appraisal for each paper.^16^ By including a range of documents the analysis identifies lessons from actual disaster experience. Each disaster is different, and these experiences may be most useful for planning in the future, rather than merely focusing on more standard disaster management practices.

Finally, the selected priorities identified by the literature analysis are compared with the components of the expert-consensus-derived WHO Toolkit. Topics that are under-represented are discussed using the literature evidence.

## Findings

Panel 1 presents a selection of health system priority areas drawn from the analysis of the core literature. These topics were then compared with the WHO Toolkit and the relevant ‘Essential Attribute’ reference in the Toolkit is identified; areas that are under-represented by the Toolkit are underlined and discussed in the next section.

**Panel 1 Health system priorities in all-hazards disaster management – with references:** EA – Essential attribute reference from the Toolkit for assessing health-systems capacity for crisis management in the WHO Regional Office for Europe 7. Underlined priorities highlight areas that are under-represented by the Toolkit, and are discussed further below

**1) Leadership and governance** • International, national and cross-boundary systems of governance, coordination and response for all-hazards disasters (Toolkit essential attribute 1a & 2a).^20^ ^21^ ^22^ ^23^ • Interwoven nature of the legal system, ethical decision-making and disaster management (EA 3d). 24 25 26 27 • Disaster management as a political issue requiring strong leadership skills (EA 4d and EA 6d). 28 29
**2) Health workforce** • Public health training in disaster management and evaluation 30 31 32 33 34 • Establishing the commitment, welfare and volunteer-credentialing of health workers (EA 15c). 35 26
**3) Medical products, vaccines and technology** • Stockpiling disaster-related medications and equipment, and their distribution (EA 17b, d & e, & EA 18 b, d & e) 29 36 37 • Required characteristics for mass vaccination and understanding previous controversies (EA 18a) 38 39 40 • Public health consequences of technology failures and impact on healthcare facilities (EA 39b & c, & EA 51) 41 42 43
**4) Health information** • Requirement to optimize the performance of health information at all stages of disaster planning (EA 21a & c, & EA 23a & b), management (EA 22b & 24) and recovery (EA 24) 44 • Awareness of research and developments in health information tools 45 • Communications – inter-agency; two-way with the public; and the role of the media as part of disaster management strategy (EA 28b,c & d) 46 28 47 23
**5) Health financing** • Health finance system consequences on disaster management effectiveness and coordination (EA 31) 48 26 • Disaster management funding issues; implications for national public - and global – health (EA 30 & 31) 38 49
**6) Service delivery** • Disaster preparedness (EA 32), acute response (EA 34) and continuing service requirements (EA 39 & EA 51) 50 40 51 • Community preparedness strategies to increase community resilience 52 53 54 55 56 57 • Surge capacity development within (EA 33) and beyond healthcare facilities 29 58 35 • Issues in adjusting to crisis standards of care and maintaining priorities (EA 32 & EA 36a & EA 38i) 27 • Safeguarding patient’s medical records and medication needs (EA 44a) during disaster 48 59

**Discussion of the literature evidence.** The WHO Toolkit provides a generic resource, enabling countries to increase the resilience of their health system to manage risks of disasters using an all-hazards approach. The qualitative analysis of the collated worldwide experience of disasters since 2000 provides a complementary literature-based approach to support and enhance the expert-consensus process used in the development of the Toolkit. The analysis has identified many priorities well represented in the Toolkit and a few areas that are under-represented.

Areas that are well represented in both the literature and the toolkit pertain to legal and governance structures for emergency management. These include prioritising national multisectoral coordination as well as health-sector emergency management. The importance of partnership-building is also recognised. Human resource development is another key component that is found in the Toolkit and widely discussed in the literature. This includes disaster training and education for health workers. Logistics and operational support for acute response delivery and the continuation of essential services during disasters are areas well represented in the Toolkit.

A selection of under-represented areas is discussed below.

**1) Leadership and governance.** Interwoven nature of the legal system, ethical decision-making and disaster management. The importance of explicitly including ethical decision-making processes in disaster management is not widespread in practice, nor mentioned in the Toolkit. For example, Thomas’ survey of disaster planners in the United States of America found that both ethical training and expertise were lacking, and concludes that there is a “risk of making many unjust and regrettable decisions... The allocation of resources and the application of control measures have enormous ethical implications, not only in the saving of lives but also in the preservation of human rights, maintenance of a functioning society, and the achievement of social justice”.^25^ The balance between individual rights versus public health benefits can be particularly difficult in times of crisis, such as the need to isolate or quarantine individuals to protect a wider population. It is important to prepare guidelines in advance so that they can be drawn upon at the time of a disaster without further delay or due consultation; transparency in decision-making is important.^24^

Disaster management as a political issue. Several authors concentrate on the involvement of political leaders due to the inherently political nature of decision-making during disasters - “Never has public health been more political or the linkages with government stronger or more demanding”.^28^ Hick argues that political authorities must be well-versed on issues, such as the allocation of scarce resources, and should be willing to trust the advice of public health and health care agencies 29. Political leaders determine what constitutes an ‘emergency’, and their laws determine issues regarding response, such as the extent of responsibility for potential or actual harms that occur as a result of the emergency.^24^

**2) Health workers.** Establishing the commitment and welfare of health workers. Levin acknowledges that health workers are at risk of becoming infected early in a pandemic and emphasises the need to address their fears and concerns in advance, highlighting the survey finding that only 48% of health workers in the United States of America would currently report to work in such a scenario.^35^ Health workers need to be confident that they will have access to all relevant protective equipment, vaccines, prophylaxis and treatments.^26^Barnett et al have explored this issue in relation to Public Health workers, and identify that rural cohorts are more willing to respond than urban based workers. They suggest that further investigation may identify factors that influence willingness to respond.^60^

**3) Health information.** Use of health information systems in disaster recovery. Tools to support disaster recovery are important. An example is the ‘Dashboard Project’ developed following Hurricane Katrina to produce demographic, economic and health indicators for the most severely affected areas, enabling ongoing disaster recovery.^44^Effective post-disaster surveillance can limit the risk of a further disasters caused by secondary causes. Infectious diseases, such as cholera or measles, can be catastrophic in vulnerable populations,^61^ and mental health problems are common in the wake of disasters.^47^ ^62^ Geographic Information Systems can be used to support the identification of populations that will require resources and new services post-disaster, enhancing information provided by other surveillance systems.^63^

Awareness of developments in health information tools. There is a need to improve health information systems so that they provide ‘rich patient data’, such as by increasing the range of data sources and undertaking real-time public health surveillance for emergency planning and response. Chretien suggests that tools should seek to enhance human judgment rather than replace it, allowing specific circumstances to be suitably analysed. There have been many developments in disaster management since 2000, but evaluations of information systems are lacking.^45^

Communications – inter-agency; two-way with the public; and the role of the media as part of disaster management strategy. The importance of crisis and risk communication is widely recognised in the literature. There are many levels at which communications must be rigorous during a crisis. For example, Dorn’s survey measures ‘connectivity’ between disaster management agencies to evaluate public health preparedness and response.^64^ Harris analysis ‘social networks’ of public health disaster planners to reveal a paucity of networking with high-level and private-sector contacts, which the authors feel are necessary for managing an effective response to a disaster.^46^

Klitzman recommends “early two-way communication (with the public)... to reduce distrust and maintain credibility”.^47^ Thus the media should be an integral part of the disaster strategy, with experts providing information.^23^ This ensures that messages are consistent, responsive, and can mitigate unnecessary anxiety.^65^

**4) Vaccinations.** Mass vaccination issues. The literature describes significant controversy over examples of mass vaccination proposals.^38^ Hupert models the likely demand on emergency health facilities as a direct result of adverse events following a mass vaccination program, finding that short vaccination campaigns would overwhelm capacity.^39^ Noji considers that mass vaccination is rarely advisable, ineffectually diverting limited resources.^40^However, each scenario must be independently judged as in certain circumstances failing to mass vaccinate against an infectious disease, such as measles, can have catastrophic consequences.^66^

Public health consequences of technology failures and impact on healthcare facilities. Prezant, using data showing that even back-up systems can be overwhelmed, suggests that public health disaster planning needs to include a wide spectrum of activities and training.^43^Bluth describes the impact of power failure on medical technologies in a hospital department, rendering services crucial to the disaster response inadequate.^41^ Public health consequences of a power failure, which may form only a part of a disaster, require contingency planning.^42^

**5) Finance systems.** Health finance system consequences on disaster management effectiveness and coordination. In times of crisis, issues relating to health finance and payment systems become acute.^48^ Disaster preparedness requires clear policies that are effective regardless of the specific funding arrangements. However, conclusions from the literature emphasise that universal access to health care without out-of-pocket payment, facilitates earlier detection of new diseases, better enables disease-control and surveillance, and minimises detriment to population health caused by delayed care.^26^ Further, in research conducted on pandemic influenza preparedness in Europe, Mournier-Jack concluded that health insurance providers should support plans to reach vaccine coverage thresholds and provide antiviral medications when required.^20^

**6) Service delivery.** Community preparedness strategies to increase community resilience. Communities need to have a comprehensive planning process, a thorough emergency operations plan, established response capability, and an ongoing surveillance and notification system for communicating emergencies.^53^ Matteson proposes the preparation of ‘home disaster kits’ and information on ‘how to prepare a shelter at home during a pandemic’. The dissemination of these public health messages, they argue, “has the additional benefit of making the larger community aware of public health preparedness activities”.^57^ These approaches are particularly important amongst communities that may lack resilience. Bouye concentrates on this issue, describing vulnerability as being as a result of “social and personal factors… confounded by system, policy and institutional factors”.^67^ A community can become empowered and act as a ‘resource’ rather than a ‘victim’ following training in search and rescue, care and shelter, emergency communications, and so on.^52^

Surge capacity development beyond healthcare facilities. Hick suggests expanding ambulatory care surge capacity to reduce pressure on hospitals, allowing them to concentrate on persons requiring higher levels of medical attention. The authors list a set of potential alternative care sites and factors to consider in the selection, while advocating the need to facilitate home family-based care.^29^ In support of home-care provision, Levin considers -“Placing large numbers of infected people in one congested (alternative care site) setting, which will probably have inadequate facilities for personal hygiene and sanitation, could serve to promote its spread and provide marginal care at best”.^35^

Issues in adjusting to crisis standards of care and maintaining priorities. The health system may become overwhelmed during a disaster requiring adjustments in standards of care and the triaged allocation of limited resources. Gostin defines crisis standards of care as “the optimal level of health care that can be delivered during a catastrophic event, requiring a substantial change in usual health care operations.” Protocols, previously prepared by experts via an evidence-based, peer-reviewed process, should be provided for use in a disaster to guide clinicians with decision-making.^27^ Hick proposes a tiered system of resources and coordination mechanisms, in which an inadequacy of resources at one level (such as community) activates the next tier of response (such as regional), and so on.^29^ There should be an established system allowing for an immediate request for assistance from neighbouring regions, and arrangements for the transfer of patients. Legal mandates may be helpful in this regard, particularly with mutual aid agreements to transfer special care patients from a nursing home, hospice or with other chronic disease such as requiring kidney dialysis.^59^

Safeguarding patient’s medical records and medication needs during disaster.Several authors focus on the need to create central databases of patient’s medical histories and medications.^48^ Arrieta highlights that patients generally lack knowledge about their medical history and thus advises that patients should be provided with summaries that can be distributed, with prescriptions linked to pharmacies with central databases.^59^

**Implications for public health, policy and disaster management multi-professionals.** Using evidence and practical experience drawn from the published literature to complement the WHO Toolkit, planners from around the world can evaluate the status of their current health system in regard to disaster management. This information can help to identify the most effective developments to be made across all levers to enhance their preparedness and resilience to all-hazards disasters.

This initial exploration provides scope for development. Future research should consider the usability and effectiveness of the health system strengthening approach across a range of low, middle and high income countries, each with their unique health system structures, environment and resource implications. The search of the core literature results in a bias towards the highly resourced United States of America, whilst the grey literature provides insights into disaster management in low-income countries. A variety of documents were included in the review, such as opinion pieces and case reports. These expose important areas of controversy and present useful, all be it specific, experiences in the field. The nature of disasters means that each incident can identify important lessons, and thus have been included in this collation.

The changing climate is increasing threats, leading to “changes in the frequency, intensity, spatial extent, duration, and timing of extreme weather and climate events, and can result in unprecedented extreme weather and climate events”.^68^ The impact is significant in large parts of the developing world where populations and health systems are already vulnerable, and disaster response management and infrastructure lacking. This work may be progressed to reduce health system vulnerabilities for these populations.

## Conclusion

Ministries and Departments of Health within countries should consider prioritising preparedness for their most likely threats, and yet plan for an all-hazards approach to disasters. Their governments have the ultimate responsibility to be able to train staff, manage a surge in need, conduct timely surveillance, prevent secondary consequences and minimise morbidity and mortality. Health agencies at the highest level need to be able to coordinate with other agencies, collaborate with other countries, communicate effectively amongst themselves and with the public, engage communities, build resilience and deliver an efficient recovery. This requires being abreast of developments in disaster management, accessing the most useful tools, with continuous planning and reflective research to identify lessons from past disasters. Previously there has been no database or collated resource of disaster management lessons, meaning that essential experience is being forgotten.

In summary, the qualitative analysis of the disaster management experience found in the published literature supports the value and content of the WHO Toolkit by identifying lessons from the actual worldwide experience of disasters since 2000. In combination, this work sets out a health system strengthening approach to building disaster management capacity. It has international relevance and applicability, as highlighted by the recent World Health Assembly’s agenda item and WHO’s commitment to developing the health system approach to disaster management. This approach will contribute to sustainable health system resilience for populations in the face of disasters.
